# Notes and Notices

**Published:** 1893-01

**Authors:** 


### NOTES AND NOTICES.

THE DIET FOR WASTING DISEASES.—In typhoid fever, phthisis, pneumonia, nervous prostration, etc., where the vitality is low and the digestive organs weak, the use of Horlick's Malted Milk as a diet has proven very beneficial, often sustaining the strength of the patient and preventing excessive emaciation and assisting in rapid recovery where recovery is possible.

This preparation is composed of rich cow's milk and an extract of malted grain, containing all the elements of nutrition in a form most easily digested. At the same time it forms a delicious drink and one acceptable to the weakest stomach. Prepared for use by simply adding water.

S. MILLS FOWLER, M. D., has removed his residence to 3269 Cottage Grove Avenue, Chicago. Hours at home 7.30 to 8.30 A. M., 5 to 7 P. M. Office 79½ Twenty-second Street. Office hours 10 A. M. to 2 P. M., except Thursdays, Saturdays, and Sundays. Lecture hours (Hering College and Hospital) Thursday 9 A. M. and 1 P. M. At Edgewater 1111 Early Avenue, every Saturday 9 A. M. to 3 P. M.

DR. FRANKLIN POWEL has removed to S. W. corner of Madison and Fifth Streets, Chester, Pa. Entrance on Fifth Street.

DR. A. R. MORGAN has removed from New York to Waterbury, Connecticut.

DR. E. P. GREGORY has removed from Waterbury, Conn., to San Antonio, Texas. Dr. Gregory, in consequence of illness in his family, has been obliged to seek a change of climate, and has therefore gone to San Antonio.

CHILDHOOD'S LOGIC.—As a touch of quaint and graceful humor few things could be better after its kind than Frances Sparhawk's little paper on "Childhood's Logic," which is printed in *Childhood* for December. The little girl and boy who talk for themselves are *real* children, and the remark made by one of them sounds so much like the outburst of a longing and repressed little soul that it strikes home: "Oh! what a good time we could have if it wasn't for God and the policemen!"

DR. WM. C. RICHARDSON, of St. Louis, Mo., has been appointed Supreme Medical Examiner to fill the vacancy caused by the death of Dr. Hugh Doherty. Dr. Richardson is eminently qualified to fill the position from education and experience as Grand Medical Examiner for the A. O. U. W. and also the Selected Knights of Missouri. A number of years ago he formulated a little book as a guide to subordinate lodge medical examiners, a copy of which should be in the hands of every one of the five thousand medical examiners for the A. O. U. W. Dr. Richardson is held in high esteem by A. O. U. W. and Select Knight fraternities of Missouri, and has been honored by positions of trust and responsibility by them, which he has filled with credit to himself and the Order.

Mr. W. B. Saunders, of 913 Walnut Street, Philadelphia, announces in preparation, for sale by subscription only, *An American Text-Book of the Medical and Surgical Diseases of Children.* By John Ashhurst, Jr., Philadelphia; Henry Dwight Chapin, New York; J. M. Da Costa, Philadelphia; G. E. de Schweinitz, Philadelphia; Albert R. Leeds, Hoboken, N. J.; Chas. K. Mills, Philadelphia; William Pepper, Philadelphia, and fifty-five other well known physicians in all parts of the United States.

Correction.—In the September number, page 382, first line, for "instructive" read INSTINCTIVE. A fatal error.

---

### FUN FOR DOCTORS.

Otherwise She Was All Right.—"Well, my dear madam, and how are you to-day?"

"O doctor! I have terrible pains all over my whole body, and it seems impossible to breathe! Of course, I can't sleep at all, and I haven't a particle of appetite!"

"But otherwise you feel all right, don't you?"—*Fliegende Blatter.*

Mrs. Gilroy.—"Here's a patent medicine man who offers a dictionary with every bottle of medicine."

Gilroy—"That's right. The dictionary is good for bad spells."—*Munsey's Weekly.*

He Wouldn't Die.—"Doctor," groaned Mr. Skinnphlint, turning his face to the wall, "it's no use! Your bill, even if I should get well, will be $100 or over!"

"Yes," replied the physician, "it will be about $100. But if you die your funeral expenses will be at least $200."

"Then I won't die!" said Mr. Skinnphlint, much disgusted.

And he didn't.—*Chicago Tribune.*

Two of a Kind.—Tom—"You look worn out, old fellow. The penalty of popularity, I suppose?"

Popular young M. D. (wearily)—"Yes, I attended two small but lively germans last night."

"Too much for one night."

"Rather. At the same house, too."

"Heavens! How odd."

"Not at all. My worthy patron, Schimmelhopfer, became the father of twins last night."

---

"But how do they do where there are no doctors?"

"People have to die without their assistance, of course."—*Pittsburgh Bulletin.*

